# Will I Have a Hangover Headache Tomorrow? A Prospective Cohort Study of the Predictors of Delayed Alcohol-Induced Headache

**DOI:** 10.3390/life15111723

**Published:** 2025-11-07

**Authors:** David García-Azorín, Lucinia Colilla-Cantalejo, Álvaro Sierra Mencía, Yésica González Osorio, Andrea Recio García, Ana Echavarría-Íñiguez, Ángel L. Guerrero

**Affiliations:** 1 Headache Unit, Department of Neurology, Hospital Universitario Río Hortega, 47012 Valladolid, Spain; dgazorin@ucm.es; 2 Department of Medicine, Faculty of Medicine, Universidad de Valladolid, 40005 Valladolid, Spain; colillalucinia@gmail.com; 3 Headache Unit, Department of Neurology, Hospital Clínico Universitario de Valladolid, 47003 Valladolid, Spain; alvarosierramencia@gmail.com (Á.S.M.); ygoinvestigacion@outlook.com (Y.G.O.); andreareciogar99@gmail.com (A.R.G.); anaechavarria93@gmail.com (A.E.-Í.); 4 Health Research Institute of Valladolid (IBioVALL), 47010 Valladolid, Spain

**Keywords:** headache disorders, migraine, alcohols, alcohol-related disorders, dehydration

## Abstract

Introduction: Alcohol-induced headaches are one of the most prevalent types of headaches. The International Classification of Headache Disorders defined them as throbbing and bilateral, and their phenotype combines characteristics of migraines and headaches secondary to low cerebrospinal fluid pressure. We aimed to evaluate the factors associated with the presence of a headache as a hangover symptom. Methods: This was a prospective cohort study, including 32 healthy individuals who voluntarily consumed alcohol and completed self-administered questionnaires during three separate alcohol consumption and hangover episodes. Results: A headache was a hangover symptom in 55/96 (57.3%) episodes. The phenotype was predominantly holocranial (94.5%), frontal (98.2%), and pressing (67.2%), with a median intensity of 6 (IQR 4–8). Headaches worsened with physical activity (100%) and had orthostatic changes (89.1%). A prior history of headaches was associated with headache occurrence (odds ratio: 3.480; 95% confidence interval (CI): 1.084 to 11.177), and headache precipitation by standing up was associated with a shorter duration (hazard ratio: 0.257; 95% CI: 0.073 to 0.901). Conclusions: Delayed alcohol-induced headaches had a migraine-like phenotype. An orthostatic pattern suggestive of a low cerebrospinal fluid pressure was associated with a shorter duration of the headache.

## 1. Introduction

Alcohol consumption produces many deleterious consequences on health. Alcohol-induced headaches are one of the most prevalent types of headaches, with a life-time prevalence of up to 72% [1]. Depending on the timing, the International Classification of Headache Disorders (ICHD) differentiates between acute and delayed alcohol-induced headache (DAIH), when the headache develops within 5–12 h after alcohol consumption, with DAIH also known as a hangover headache. According to the ICHD, the phenotype of DAIH must be throbbing in quality, must be bilateral in location, and must worsen with physical activity [2].

The phenotype of DAIH combines clinical features of migraines and low cerebrospinal fluid (CSF) pressure headaches [3]. The migraine-like presentation may be explained by the vasodilator effect of alcohol [4]. Among the three phases involved in alcohol pharmacokinetics [5], absorption may be affected by different situations concerning the gastrointestinal tract, distribution is commonly quick and homogeneous, and elimination has a limited capacity. These variables, as well as possible individual predisposition [6], might lead to the delayed onset of a headache. The low-CSF pattern may be caused by the inhibition of antidiuretic hormone secretion induced by alcohol, causing systemic and intracranial dehydration [7,8].

It is a common belief that some factors, such as proper hydration or food consumption, may prevent the incidence of headaches following alcohol consumption; however, this has not been demonstrated with scientific evidence [9]. In the present study, we aimed to evaluate which factors were associated with the presence of a headache as a hangover symptom in a cohort of individuals that voluntarily consumed alcohol. We prospectively collected data related to the intake of alcohol and other beverages or food, as well as systematic descriptions of hangover syndrome and the headache phenotypes and durations.

## 2. Materials and Methods

This was an observational analytic study with a prospective cohort design. The study design and reporting were based on the Strengthening the Reporting of Observational Studies in Epidemiology (STROBE) [10] guidelines. The study was conducted in accordance with the principles of the Declaration of Helsinki [11], and the Valladolid East Ethics Review Board approved the study (code: PI21-2423TFG). The study was performed between 6 August and 26 September 2021.

### 2.1. Eligibility Criteria

Participants were included if they (1) signed an informed consent form; (2) were aged between 18 and 40 years; and (3) planned to consume alcohol voluntarily at an expected amount higher than the tolerable upper limit intake.

Participants were excluded if they (1) had systemic, neurological, or psychiatric disorders that made it difficult to assess the episode and the headache phenotype description; (2) had another incident headache that was better accounted for by another secondary headache disorder (i.e., 5.1 acute headache attributed to traumatic injury to the head); (3) consumed illicit drugs that hampered the assessment of the episode and the headache phenotype description; (4) were exposed to drugs with headache as an adverse event; (5) had insufficient performance of the Spanish language.

### 2.2. Recruitment and Sampling

The study was advertised to Valladolid University students by using a convenience non-probabilistic recruitment and snowball sampling.

### 2.3. Study Intervention

The study consisted of three different questionnaires. The first questionnaire was administered upon enrolment, while each participant completed the second and third questionnaires during each of three isolated alcohol consumption episodes.

First, a researcher conducted an in-person interview and administered a structured hetero-administered questionnaire, based on previous studies (Supplementary Materials) [3], which included demographic variables such as age, sex, weight, height, body mass index (BMI), and occupation. Prior medical history included a prior history of headaches in the patient or their relatives and the specific diagnosis and source of the diagnosis. Prior history of neurological, gastroenterological, hepatic, and psychiatric diseases and illicit drug use were assessed. Prior history of alcohol-induced headaches was also assessed, including the usual amount of alcohol consumed during the weekdays and over the weekend. Sleep was assessed, including the habit of napping.

Second, participants completed a structured self-administered questionnaire during (i.e., prospectively) the alcohol consumption episodes (Supplementary Materials), which assessed the type and number of alcoholic beverages, non-alcoholic beverages, and food consumed. Participants kept and completed a log as they were drinking. The total number of hours of sleep was evaluated. To avoid a potential confounding factor, grams of alcohol as well as non-alcoholic beverages were corrected considering body mass index.

Third, the day after the alcohol consumption episode, participants completed another structured self-administered questionnaire (Supplementary Materials), which prospectively collected information about the presence of a headache and other hangover symptoms hourly for one day, and the specific phenotype of headache. The need for acute medication was assessed.

### 2.4. Statistical Analysis

Qualitative and ordinal variables are presented as frequencies and percentages and quantitative variables as means and standard deviations (SD) in the case of normal distribution, or medians and inter-quartile ranges (IQR) otherwise. Kolmogorov–Smirnov tests were used to assess the normality of the distributions. For hypothesis testing, chi-squared tests, independent sample Student’s *t*-tests, and Mann–Whitney U tests were used. All tests were two-tailed, and the *p* value threshold was set as <0.05.

To evaluate which variables were associated with the presence of a headache, a univariate logistic regression analysis was performed. All variables that presented a *p* value <0.2 were included in a multivariable logistic regression analysis. Results are presented as odds ratios (ORs) and 95% confidence intervals (CIs).

To evaluate which variables were associated with a more prolonged duration of the headache, a Cox regression was employed. Firstly, log-rank tests were used, and all the variables that had a *p* value < 0.2 were included in a multivariate Cox regression analysis. Results are expressed as hazard ratios (HRs) and 95% CIs.

Due to the exploratory nature of the study, an a priori power calculation was not performed, and a sample of 30 participants was considered adequate. Statistical analyses were conducted with SPSS v26.0 (IBM Corp., Armonk, NY, USA).

## 3. Results

A total of 32 participants were screened, and all were eligible. All participants completed questionnaires for 3 valid episodes of alcohol consumption, for a total of 96 episodes assessed. The participants had a median age of 22.5 (IQR: 21–24) years, and 23 (71.9%) were female. Regarding occupation, 20 (62.5%) were students, 10 (31.3%) were full-time employees, and two (6.3%) were unemployed.

### 3.1. Prior Medical History

A prior history of primary headaches was reported by 15 (46.9%) participants, with 8/15 (53.3%) having been diagnosed by a physician. Among these, the diagnosis was a migraine in 6/8 (75%) and a tension-type headache in 2/8 (25%). These patients had been diagnosed by a neurologist in 6/8 (75%) cases and by a primary care physician in 2/8 (25%). A family history of headaches was reported in 13 (40.6%) of the cases. Participants had a prior history of gastrointestinal disorders in six (18.8%) cases, psychiatric disorders in three (9.4%) cases, and kidney disorders in two (6.3%) cases, and there were no cases of hepatic disorders.

### 3.2. Alcohol Consumption and Sleeping Habits

Seven (21.9%) participants reported regular alcohol consumption, with beer being the consumed beverage in all cases. All participants reported that the most frequently consumed alcoholic beverages on the weekends were spirits, with a median of 210 (IQR: 126–252) grams of pure alcohol. Seven (21.9%) participants reported smoking tobacco regularly, and one participant reported occasionally smoking cannabis.

All participants declared prior episodes of hangover, estimating that it occurred approximately 70% (IQR: 52.9–90%) of the times that they consumed alcohol over the weekend. Concerning sleeping habits, participants reported a median time of usual sleep of 8 (IQR: 7–8.37) hours per day, with a range between 5.5 and 9 h. Four (12.5%) participants took a short nap after lunch regularly.

### 3.3. Alcohol Consumption During the Studied Episodes

The amount of alcohol consumed during the 96 studied episodes had a median of 252 (IQR: 192–336) grams of pure alcohol per episode, with a range between 106 and 714 g. Non-alcoholic beverages were consumed during 30/96 (31.3%) episodes, with a median volume of 660 (IQR: 330–990) milliliters; carbonated drinks were the most frequently consumed beverage in 26/30 (86.7%) cases, followed by water in 4/30 (13.3%) cases. Participants ate some amount of food during the episode in 44/96 (45.8%) episodes. The median number of hours of sleep during the studied episodes was 6.1 (IQR: 5.3–7.0) hours.

### 3.4. Frequency and Phenotype of Headache

A headache was reported as one of the hangover symptoms in 55/96 (57.3%) episodes. Among headache sufferers, headaches were present on waking in 38/55 (69.1%) cases and had a delayed onset in 17/55 (30.9%) cases. The median duration of the headache the day after alcohol consumption was 2 (IQR: 2–3) hours, with a range between 1 and 7 h.

**Figure 1:**
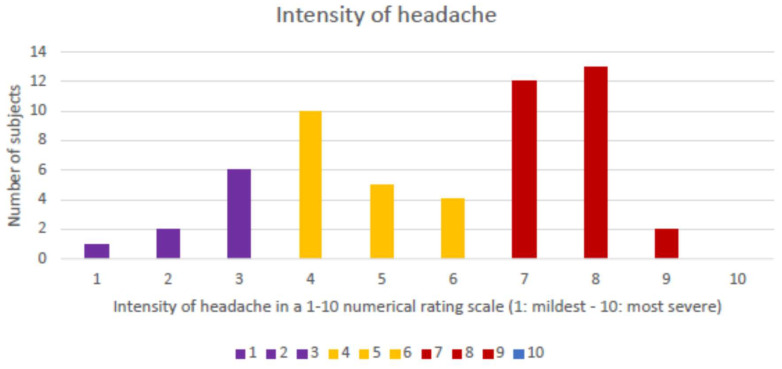
Histogram of number of participants by level of intensity of the headache.

The headache was reported as holocranial in 52/55 (94.5%) cases and hemicranial in 3/55 (5.5%) episodes, corresponding to 3 patients with a previous history of migraine. The proportion of participants that reported pain in each of the different topographies was 54/55 (98.2%) for frontal, 20/55 (36.4%) for temporal, 2/55 (3.6%) for parietal, and 1/55 (1.8%) each for occipital or periocular. The quality of the headache pain was described as pressing in 37/55 (67.2%), stabbing in 15/55 (27.3%), and throbbing in 3/55 (5.5%). The median intensity of the headache, measured according to a numeric rating scale (1: mildest pain–10: the most severe imaginable pain), was 6 (IQR: 4–8). Figure 1 presents the number of participants that reported each level of intensity.

Participants reported a worsening of the headache with physical activity in all episodes, graded as mild in 2/55 (3.6%), moderate in 40/55 (72.7%), and severe in 13/55 (23.7%) episodes. Participants described that the headache interfered with their usual activity in 49/55 (89.1%) episodes, being graded as moderate in 42/55 (76.4%) and severe in 7/55 (12.7%) episodes. Data regarding the associated symptoms are included in Supplementary SII of the Supplementary Data. There were no statistically significant differences in the frequency of any symptoms between participants with and without a headache.

### 3.5. Orthostatic Pattern

Participants reported headache precipitation/worsening with orthostatic changes in 49/55 (89.1%) episodes, including a precipitation of the headache when changing from lying down to a standing position in 38/55 (65.5%), worsening when changing from lying down to a standing position in 32/55 (58.2%), and improvement of the headache when lying down in 34/55 (61.8%) cases.

### 3.6. Symptomatic Treatment

Acute medications to alleviate the headache were used in 26/55 (47.3%) episodes, including non-steroidal anti-inflammatory drugs in 18/26 (69.2%), paracetamol/acetaminophen in 7/26 (26.9%), and metamizole in 1/26 (3.8%) cases.

### 3.7. Predictors of Headache

**Figure 2:**
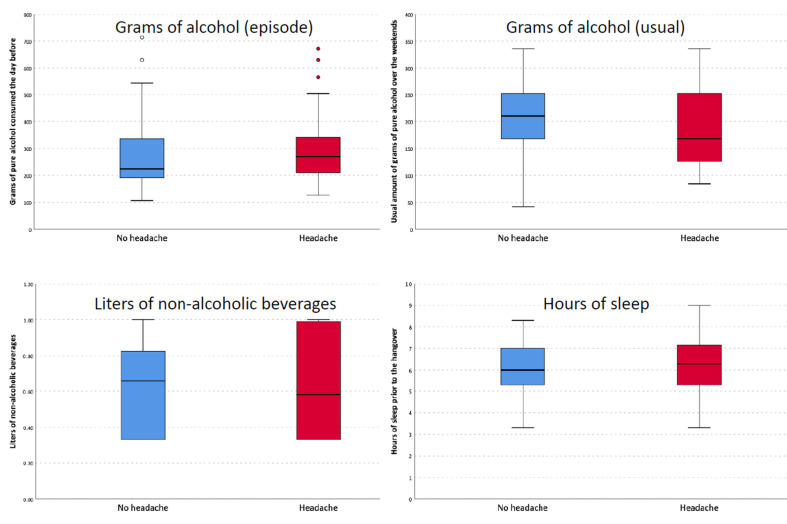
Differences in grams of alcohol, non-alcoholic beverages, and hours of sleep between patients with and without headache.

There were no differences in the amount of alcohol consumed the night prior to the episode (g), the usual amount of alcohol consumed (g), the volume of non-alcoholic beverages consumed (L), or the hours of sleep between participants who had headaches and the rest of the sample (Figure 2). In the univariate analysis (Supplementary Materials), the variables that were associated with the incidence of headache were a prior history of headaches (OR: 3.437; 95% CI: 1.347–8.775) and the average amount of pure alcohol (g) regularly consumed on weekdays (OR: 0.957; 95% CI: 0.922–0.993). The presence of a headache was also associated with a more prolonged duration of thirst as a hangover symptom (OR: 1.506; 95% CI: 1.086–2.088), a more prolonged duration of the difficulty in thinking or speaking normally (OR: 2.566; 95% CI: 1.254–5.247), and the presence of difficulty in reading or writing normally (OR: 2.830; 95% CI: 1.250–6.406). In the multivariate analysis (Table 1), the only variable that remained statistically significant was a prior history of headaches (OR: 3.480; 95% CI: 1.084–11.177).

### 3.8. Predictors of a More Prolonged Headache

**Figure 3:**
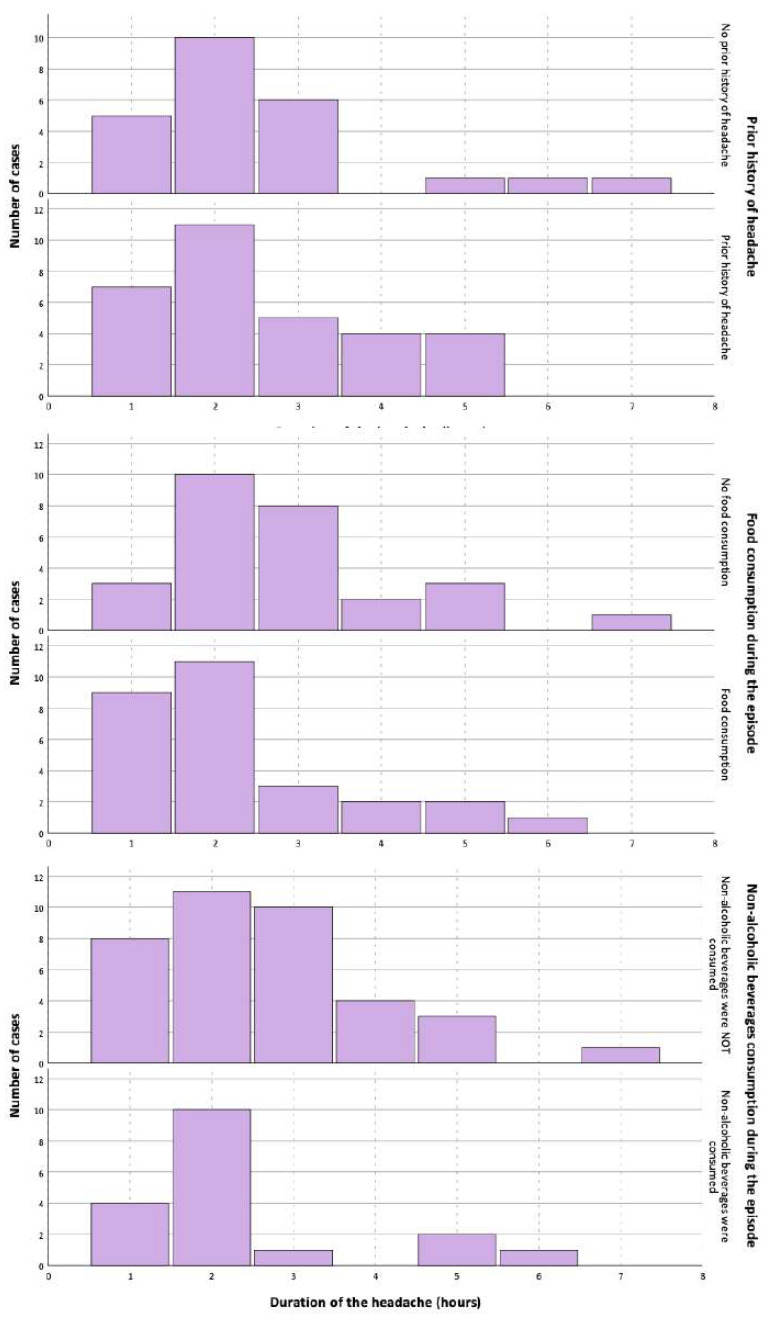
Differences in the duration of headache depending on the presence of prior history of headache, food consumption, or non-alcoholic beverage consumption.

There were no statistically significant differences in the duration of the headache between participants with or without a prior history of headaches, with or without food consumption during the studied episode, or who did or did not drink non-alcoholic beverages during the episode (Figure 3). In the univariate Cox regression (Supplementary Materials), the variables that were associated with the duration of the headache were headache intensity (HR: 0.848; 95% CI: 0.745–0.964), the presence of photophobia (HR: 0.512; 95% CI: 0.294–0.892), the presence of dizziness/vertigo (HR: 0.472; 95% CI: 0.264–0.844), the duration of phonophobia (HR: 0.665; 95% CI: 0.481–0.921), the duration of photophobia (HR: 0.690; 95% CI: 0.482–0.990), the duration of dizziness/vertigo (HR: 0.669; 95% CI: 0.458–0.975), and the duration of the inability to read/write normally (HR: 0.634; 95% CI: 0.458–0.975). In the multivariate analysis, the only variable that remained statistically significant was the precipitation of the headache by standing up (HR: 0.257; 95% CI: 0.073–0.901).

### 3.9. Association Between Key Variables and the Presence or Duration of Headache

Table 2 summarizes the results of the univariate logistic regression and Cox regression regarding the possible predictors of headache occurrence and those that were associated with a more prolonged duration.

## 4. Discussion

A hangover is a prevalent condition with a major impact on health. In the present study, we prospectively evaluated the presence, predictors, duration, and phenotype of DAIH in 32 participants who voluntarily consumed alcohol 3 times each, accounting for a total of 96 hangover episodes. The main findings were that headaches occurred in 57% of the studied episodes, and headache incidence was mainly associated with a prior history of headaches.

The clinical phenotype of DAIH was a holocranial (95%) headache, with a predominantly frontal (98%) and temporal (36%) location, with a pressing (67%) or stabbing (27%) quality and a moderate intensity. Phonophobia (95%), photophobia (88%), and nausea (19%) were common [3,12,13,14]. A worsening with physical activity occurred in all episodes. These findings are in line with prior studies [3,12,13] but contradict the ICHD criteria (2), since a throbbing quality of the pain was only reported by 5% of participants. Some participants may have misinterpreted regularly repeating stabs as stabbing, instead of throbbing, but, even so, this would only account for 34% of episodes. Most secondary headache disorders are bilateral in location, while the unilateral topography of some primary headache disorders remains an enigma.

In the present study, the frequency of other typical symptoms of migraines, such as photophobia, phonophobia, or worsening with physical activity, were higher than in prior studies [3,12,13], which may be explained by a more accurate evaluation, based on the prospective design and structured, systematic evaluation of each symptom over time. Also, almost half of the participants had a prior history of headaches, with DAIH being three times more frequent in participants with a prior history of headaches. We interpret that the main reasons why the clinical phenotype was similar to that of a migraine included the individual predisposing biology [15,16], the exposure to a vasodilating agent (alcohol), and the meningeal impairment caused by dehydration and intracranial hypotension [17,18,19]. On the other hand, the high doses of alcoholic intake could also explain the presence of a headache, since low amounts of alcohol were not associated with an increased risk of having a migraine episode in patients with migraines [20].

In line with prior studies [3,12,13], low-CSF pressure headache features (headache precipitation/worsening with orthostatic changes) were common and more frequent in the present study, where nine out of ten participants reported these. Ethanol inhibits antidiuretic hormone secretion [21], which causes a hydration-resistant dehydration [8]. The antidiuretic hormone stimulates the insertion of aquaporins in the kidney tubules, causing solute-free water reabsorption through tubular cells [22]. The total urine volume and urine osmolarity were not controlled or assessed in this study; however, there is an inverse relationship between urine osmolarity and urine flow rate [8]. In our study, we did not observe that participants who drank more non-alcoholic beverages suffered from DAIH less frequently or had it in a milder form, but, when there was a headache precipitation by sitting upright or standing, the duration was 75% shorter, which could reflect a different pathophysiological mechanism.

One of the strengths of this study is the systematic evaluation of the possible DAIH predictors and the possible factors that may modulate its duration. To date, several drugs and non-pharmaceutical compounds have been used in the treatment of DAIH, including tolfenamic acid, L-cysteine, pyritinol, red ginseng, clove extract, *Hovenia dulcis* fruit extract, or Korean pear juice, generally with a poor quality of evidence [23]. We tried to validate the popular belief that eating in conjunction with alcoholic beverages may minimize the odds of having a hangover; however, our findings did not support this. This could be related to the participants’ age and good health, the sample size, or the shorter hangover duration (median of two hours) than has been reported in other studies [3,12,13]. Participants who ate prior to sleeping had a shorter duration, albeit the differences were not statistically significant. In addition, hunger was reported as a symptom of the hangover in 95% of episodes, but this could potentially be either a cause or a consequence of the hangover [24]. Prior studies have observed that an adequate zinc and nicotinic acid intake is associated with a milder hangover [25].

The global burden of disease attributable to alcohol consumption is notable, particularly in Eastern, Central Europe, and North America, where it accounts for 13.8%, 9.4%, and 4.5% of disability-adjusted life-years [26], respectively. The prevalence of alcohol consumption reaches up to 26% between 15 and 19 years and 42% percent between 20 and 24 years of age [27], with numbers that have progressively increased since 2007 [28]. The mortality attributed to alcohol was higher than that of tuberculosis, HIV, or diabetes, mostly secondary to injuries, digestive diseases, cardiovascular diseases, mental disorders, or self-harm and interpersonal violence [26,29].

The present study has notable limitations. First, the sample size was modest, with only 32 participants and 96 alcohol consumption episodes. Prior studies with larger sample sizes detected differences, which were small, suggesting that the consequences of alcohol and the hangover phenotype may be affected by several factors [30]. The Hawthorne effect may have biased the results, since participants knew the purpose of the study and were instructed to report their activities and symptoms. The duration of the headache was shorter than that reported in other studies [3], which could be related to the participants’ young age or the use of acute medications in 47% of cases. There is no population at a higher risk of memory bias than individuals exposed to alcohol. However, the prospective design of this study and systematic evaluation attempted to minimize the risk of recall bias. All participants completed the study and provided valid data, which permitted the adjustment for several relevant confounders. Larger studies may be able to delineate additional predictors of DAIH that this study was unable to see, contributing to the knowledge of how to avoid or minimize DAIH and thus reduce health-related disability and the economic burden caused by absenteeism and presenteeism.

## 5. Conclusions

DAIH occurred in 57% of alcohol consumption episodes. It was three times more frequent in participants with a prior history of headaches. The clinical phenotype showed a holocranial location with frontal predominance, a pressing quality, and a moderate intensity. The typical symptoms of a migraine were frequent, including photophobia, phonophobia, nausea, vomiting, and worsening with routine physical activities. Most patients reported an orthostatic pattern suggestive of low-CSF pressure.

## Figures and Tables

**Table 1:** Multivariate logistic regression of the variables that were associated with the presence of headache as a symptom of hangover.

Variable	Odds Ratio	95% Confidence Interval	*p* Value
Prior history of headache	3.480	1.084–11.177	0.036
Usual hours of sleep	1.150	0.669–1.976	0.613
Weekday alcohol intake	0.968	0.924–1.015	0.176
Photophobia	1.818	0.609–5.429	0.284
Concentration difficulties	1.607	0.512–5.045	0.416
Malaise, abdominal pain, or diarrhea	1.900	0.599–6.026	0.276
Duration of photophobia	0.929	0.370–2.334	0.875
Duration of osmophobia	2.959	0.889–9.844	0.077
Duration of asthenia	1.354	0.927–1.978	0.116
Duration of thirst	1.392	0.936–2.072	0.103
Duration of hunger	0.980	0.679–1.413	0.912
Duration of inability to think or speak normally	1.018	0.367–2.823	0.972
Duration of inability to read or write normally	1.051	0.311–3.556	0.936
Duration of preference for lying down	0.796	0.550–1.153	0.228

**Table 2:** Summary of variables associated with the presence or more prolonged duration of headaches.

Variable	Odds Ratio (95% CI)	Hazard Ratio (95% CI)
Prior history of headache	3.437 (95% CI: 1.346–8.775)	1.226 (95% CI: 0.714–2.103)
Usual weekday alcohol consumption	0.957 (95% CI: 0.922–0.993)	0.997 (95% CI: 0.968–1.027)
Usual weekend alcohol consumption	0.997 (95% CI: 0.992–1.002)	1.001 (95% CI: 0.997–1.005)
Non-alcoholic beverage consumption	1.176 (95% CI: 0.489–2.827)	1.141 (95% CI: 0.643–2.024)
Eating during alcohol consumption	1.464 (95% CI: 0.648–3.310)	1.366 (95% CI: 0.799–2.335)
Usual time spent sleeping	1.363 (95% CI: 0.902–2.061)	1.126 (95% CI: 0.847–1.497)
Time slept the night before the episode	1.136 (95% CI: 0.831–1.553)	1.057 (95% CI: 0.881–1.267)

## Data Availability

Datasets and additional information are available upon reasonable request to the corresponding author.

## Supplementary Materials

The following supporting information can be downloaded at: https://www.mdpi.com/article/10.3390/life15111723/s1, Supplementary SI: Hetero-administered questionnaire; Supplementary SII: Auto-administered questionnaire; Supplementary Table S1: Total duration of symptoms; Supplementary Table S2: Frequency and duration of symptoms within the entire study sample; Supplementary SIII: Self-administered questionnaire for data collection total duration of symptoms; Supplementary Table S3: Univariate logistic regression of variables associated with the appearance of headache; Supplementary Figure S1: Amount of pure grams of alcohol within episodes where headache was present versus not present; Supplementary SIV: Supplementary Table S4: Cox regression analysis of the variables associated with a more prolonged duration of the headache; Supplementary SV: Supplementary Table S5: Multivariate Cox regression analysis: variables associated with headache duration (HR: Hazard Ratio; CI: Confidence Interval).

## Abbreviations

The following abbreviations are used in this manuscript.

ICHD	International Classification of Headache Disorders
DAIH	Delayed alcohol-induced headache
CSF	Cerebrospinal fluid
STROBE	Strengthening the Reporting of Observational Studies in Epidemiology
BMI	Body mass index
SD	Standard deviation
IQR	Inter quartile range
OR	Odds ratio
CI	Confidence interval
HR	Hazard ratio
