# Supplementary material for: Will I Have a Hangover Headache Tomorrow? A Prospective Cohort Study of the Predictors of Delayed Alcohol-Induced Headache
Source: Life (Basel). 2025 Nov 7;15(11):1723. doi: 10.3390/life15111723 (PMC12653520; doi:10.3390/life15111723)
Supplement: Supplementary file 1 [file life-15-01723-s001.zip › life-3970766-supplementary.pdf]

Will I have a hangover headache tomorrow? A prospective cohort study of the predictors of delayed alcohol-induced headache

SUPPLEMENTARY MATERIALS:

SUPPLEMENTARY SI

HETERO-ADMINISTERED QUESTIONNAIRE

A) DEMOGRAPHIC VARIABLES.

1. Sex
   a. Female, male, non-binary, other (please specify)
2. Date of birth.
3. Weight (kilograms).
4. Height (centimeters).
5. Occupation.
   a. Student
      i. Year of study
      ii. Degree
   b. Employed
   c. Unemployed
   d. Housework

B) PRIOR MEDICAL HISTORY.

6. Do you have occasional headaches? (Apart from alcohol-induced headaches)

a. Yes/No

7. Have you ever been evaluated for the headache?

   a. Yes/No

8. Does alcohol-induced headache remind you your usual headache?

   a. Yes/No

9. If the answer to the above question is yes, do you have any diagnosis? (please mark all that apply)

   a. Migraine

   b. Tension-type headache

   c. Others (please specify)

   d. I don´t have any diagnosis

10. Who made the diagnosis?

11. Answer (yes/no) to the following statements about your usual headache:

- Do you have frequent or severe headaches?
- Does headache usually last more than 4 hours

- In general, when you have a headache, do you feel nausea?
- Does light or noise bother you when you have a headache?
- Does headache limit any of your physical or intellectual activities?

12. Do you have any prior history of:
    a. … Neurological diseases? (please specify)
    b. … Digestive diseases? (please specify)
    c. … Psychiatric disorders? (please specify)
    d. … Liver diseases? (please specify)
    e. … Kidney diseases? (please specify)
    f. … Other relevant diseases? (please specify)
13. Do you take any medication regularly?
    a. Yes (please specify)/No.
14. Are you smoker? Yes/No.
15. Do you use illicit drugs?
    a. Yes (please specify)/No.
16. Had you ever experienced hangover before?
    a. In case of affirmative answer, please indicate the estimated percentage of times when you have hangover over the total number of times you drink alcohol.
17. What time do you usually wake up and go to sleep?
    a. Time you wake up: (time)
    b. Time you go to sleep (time)
18. Do you usually sleep a nap? Yes/No.

19. How much alcohol do you usually drink during the week?
20. How much alcohol do you usually drink on the weekend?

*To collect the answers to these last two questions, participants marked the estimated amount based on the first table of Questionnaire II, to familiarize the participant with it and obtain an approximate estimate of their usual alcohol consumption.

Questionnaire about the usual amount of alcohol.

MARK ACCORDING TO THE INITIAL LETTERS: W-Water, B-Beer, DS-Dark Spirits (rum, whiskey, bourbon…), NAB –Non-Alcoholic Beer, WS-White Spirits (gin, vodka), C-Calimocho (wine and coke), SD-Soft Drinks (alcohol-free), SRW-Summer Red Wine with Lemon, W-Wine, J-Juice.

QUANTITY	MONDAY	TUESDAY	WEDNESDAY	THURSDAY
Shot				
Draft beer				
Bottle 0,2 L				
Tall glass				
Bottle 0,33 L				
Jar				
Bottle 0,5 L				
Big glass.				
1 Liter				
Other (specify)				

QUANTITY	FRIDAY	SATURDAY	SUNDAY
Shot			
Draft beer			
Bottle 0,2 L			
Tall glass			
Bottle 0,33 L			
Jar			
Bottle 0,5 L			
Big glass			
1 Liter			
Other (specify)			

C) FAMILY HISTORY.

21. Do you have family history of headache in your close relatives (parents, children, siblings, grandparents)?

a. Yes (indicate relationship and diagnosis if any)

b. No

SUPPLEMENTARY SII

SELF-ADMINISTERED QUESTIONNAIRE FOR DATA COLLECTION

Time of first alcoholic beverage:

MARK ACCORDING TO THE INITIAL LETTERS: W-Water, B-Beer, DS-Dark Spirits (rum, whiskey, bourbon…), NAB –Non-Alcoholic Beer, WS-White Spirits (gin, vodka), C-Calimocho (wine and coke), SD-Soft Drinks (alcohol-free), SRW-Summer Red Wine with Lemon, W-Wine, J-Juice.

Quantity	1st Hour	2nd Hour	3rd Hour	4th Hour	5th Hour	6th Hour	7th Hour	8th Hour
Shot 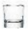								
Draft Beer 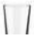								
Bottle 0,2 L								
Tall glass 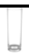								
Bottle 0,33 L 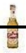 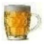								
Jar								
Bottle 0,5 L 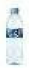								
Big glass								
1 Liter 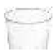								
Other (specify)								
Food (specify)								

Quantity	9th Hour	10th Hour	11th Hour	12th Hour	13th Hour	14th Hour	15th Hour	16th Hour
Shot 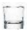								
Draft Beer 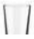								
Bottle 0,2 L								
Tall glass								
Bottle 0,33 L 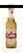								
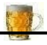 Jar								
Bottle 0,5 L 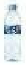								
Big glass								
1 Liter 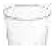								
Other (specify)								
Food (specify)								

SLEEP AND FOOD CONSUMPTION:

	ALCOHOL INTAKE DAY	DAY FOLLOWING THE ALCOHOL INTAKE
Time you woke up in the morning		
Breakfast time		
What did you have for breakfast?		
Lunch time		
What did you have for lunch?		
Nap time and duration		
Dinner time		
What did you have for dinner?		
Bedtime		

Data collection table about hangover headache to be completed by the participant the day after the alcohol intake. The participant was instructed to mark all the statements that were present in the time interval from when he/she woke up until he/she went to sleep (range 19-24ºh).

**NAME AND DATE OF FILLING THIS TABLE:**	**1º H**	**2º H**	**3º H**	**4º H**	**5º H**	**6ªH**	**7ªH**	**8ºH**	**9ºH**	**10ºH**	**11ºH**	**12º H**	**13-18º H**	**19-24º H**
1. Intensity of your headache (0-none, 10-maximum). If you mark 0 go directly to question 14.														
2. Where is the pain?														
- On both sides of the head.														
- Only on one side of the head.														
3. When did the headache appear?														
- At the time of awakening.														
- After a few hours of waking														

up.														
4. Where is the pain located? (mark all the areas that hurt)														
- Frontal (1)														
- Temporal (2)														
- Parietal (3)														
- Occipital (4)														
- Periocular (5)														
- Cervical (6)														
5. Does the pain radiate in any direction?														
- Yes (specify the direction)														
- No														
6. How would you describe the pain? (mark all that may apply)														
- Pressing														
- Stabbing														
- Throbbing														
- Burning														
- Electric (shock)														
7. Does head movement, “rattling” (movement similar to that of the cars of a freight train) make the pain worse?														
- Mildly, moderately or severely														

8. Does the headache appear when …?														
- You stand up (Yes/No)														
- You lie down (Yes/No)														

9. Does headache get worse when …?														
- You stand up (Yes/No)														
- You lie down (Yes/No)														
10. Does headache improve when …?														
- You stand up (Yes/No)														
- You lie down (Yes/No)														
11. Does the headache interfere with your daily activities?														
No (N), mildly(M), moderately (MO), severely (S)														
12. Do you feel discomfort when you touch the skin in the area of your pain?														
- Yes/No														
13. What drug do you take to get rid of it? Mark the moment you take it by making a cross in the corresponding box.														
- Paracetamol/Acetaminophen														
- Ibuprofen/Naproxen/Dexketoprofen														
- Metamizol														
- Triptan														
- Others (please specify)														
- No drugs (indicate what you take)														

14. Do the sounds bother you?														
No (N), mildly(M), moderately (MO), severely (S)														
15. Do the lights bother you?														
No (N), mildly(M), moderately (MO), severely (S)														
16. Do the smells bother you?														
No (N), mildly(M), moderately (MO), severely (S)														
17. Do you have nausea?														
- Yes/No														
18. Have you vomited?														
- Yes/No														
19. Do you have watery eyes, red eyes or nasal congestion?														
- Yes/No														
- If the answer is yes, please indicate if it is U-Unilateral or B-Bilateral.														
20. Mark if you have any of the following symptoms (Yes/No)														

- Ear fullness														
- Asthenia/tiredness														
- Dizziness or vertigo (things going around)														
- Restlessness														
- Pallor														
- Stiff neck or difficulty to move it														
- Blurred vision														
- Thirst														
- Hunger														
- Restlessness														
- Sensitive or any alteration in emotions														
- Difficulty to think or speak														
- Difficulty to read or write														
- Difficulty to														

concentrate														
- Preference for lying down														
- Sweating														
- Flushing or facial redness														
- Ptosis (droopy eyelids)														
- Miosis (small pupils)														
- Mydriasis (large pupils)														
21. Other symptoms (please specify)														

Table S1. TOTAL DURATION OF SYMPTOMS

This table will be filled in by the author of the final degree project, considering the data collected in the previous table by the study participants. The numbering corresponds to the order of the symptoms described in the previous table.

	TOTAL DURATION
1	
2	
3	
4	
5	
6	
7	
8	
9	
10	
11	
12	
13	
14	
15	
16	
17	
18	
19	

	TOTAL DURATION
20. Following symptoms	
- Ear fullness	
- Asthenia	
- Dizziness or vertigo	
- Restlessness	
- Pallor	
- Stiff neck	
- Blurred vision	
- Thirst	
- Hunger	
- Irritability	
- Sensitive or any alteration in emotions	
- Difficulty to think or speak	
- Difficulty to read or write	
- Difficulty to concentrate	
- Preference for lying down	
- Sweating	
- Flushing or facial redness	
- Ptosis (droopy eyelids)	
- Miosis (small pupils)	
- Mydriasis (large pupils)	
21. Other symptoms	

Table S2: Frequency and duration of symptoms within the entire study sample, within participants who had headache as a symptom of hangover and those who did not experience headache.

	All studied episodes (n=96)	Duration within all episodes (n=96)	Episodes with headache (n=55)	Duration of episodes with headache (n=55)	Episodes without headache (n=41)	Duration of episodes without headache (n=41)	*P* value
Photophobia	85/96 (88.5%)	2 (1-2)	50/55 (90.9%)	2 (1-2)	35/41 (85.4%)	1 (1-2)	0.195
Phonophobia	91/96 (94.8%)	2 (1-3)	54/55 (98.2%)	2 (1-3)	37/41 (90.2%)	1 (1-2)	0.147
Osmophobia	28/96 (29.1%)	0 (0-1)	18/55 (21.7%)	0 (0-1)	10/41 (24.4%)	0 (0-0.5)	0.488
Nausea	18/96 (18.8%)	0 (0-0)	12/55 (21.8%)	0 (0-0)	6/41 (14.6%)	0 (0-0)	0.372
Vomiting	11/96 (11.5%)	0 (0-0)	8/55 (14.5%)	0 (0-0)	3/41 (7.3%)	0 (0-0)	0.271
Cranial autonomic symptoms (tearing, eye redness, runny nose,	59/96 (61.5%)	1 (0-2)	34/55 (61.8%)	1 (0-1)	25/41 (61.0%)	1 (0-1)	0.933
	Unilateral 1/59		Unilateral 1/34		Unilateral 0/25		0.387

nasal fullness)	(1.7%) Bilateral 58/59 (98.3%)		(2.9%) Bilateral 33/34 (97.1%)		(0%) Bilateral 25/25 (100%)		
Ear Fullness	2/96 (2.1%)	0 (0-0)	1/55 (1.8%)	0 (0-0)	1/41 (2.4%)	0 (0-0)	0.83 3
Interference with activity	49/96 (51.1%)	2 (1-2)	54/55 (98.2%)	2 (1-2)	27/41 (65.8%)	0 (0-0)	0.72 6
Thirst	95/96 (99.0%)	5 (4-5)	54/55 (98.2%)	5 (4-6)	41/41 (100%)	1 (1-1)	0.38 5
Hunger	91/96 (94.8%)	3 (2-4)	52/55 (94.5%)	3 (2-5)	39/41 (95.1%)	1 (1-1)	0.90 0
Asthenia	89/96 (92.7%)	4 (3-5)	52/55 (94.5%)	4 (4-6)	37/41 (90.2%)	1 (1-1)	0.42 3
Clinophylia	85/96 (88.5%)	3 (2-4)	49/55 (89.1%)	3 (2-4)	36/41 (87.8%)	1 (1-1)	0.84 5
Difficulty concentrating	59/96 (61.5%)	1 (1-1.75)	37/55 (67.3%)	1 (1-2)	22/41 (53.7%)	1 (0-1)	0.17 5
Digestive symptoms	59/96 (61.5%)	1 (0-2)	37/55 (67.3%)	1 (0-2)	22/41 (53.7%)	1 (0-1)	0.17 5
Dizziness / vertigo	45/96 (46.9%)	0 (0-1)	28/55 (50.9%)	0 (0-1)	17/41	(41.5%)	0.35 9
Altered mood /	31/96	0 (0-1)	16/55	0 (0-1)	15/41	0 (0-1)	0.43

feelings	(32.3%)		(29.1%)		(36.6%)		7
Restlessness	26/96 (27.1%)	0 (0-1)	15/55 (27.3%)	0 (0-1)	11/41 (26.8%)	0 (0-1)	0.96 1
Irritability	26/96 (27.1%)	0 (0-1)	13/55 (23.6%)	0 (0-0)	13/41 (31.7%)	0 (0-1)	0.37 9
Difficulty thinking or speaking	24/96 (25.0%)	1 (1-1)	15/55 (27.3%)	1 (1-1)	9/41 (22.0%)	0 (0-0)	0.55 1
Difficulty reading or writing	18/96 (18.8%)	1 (0.25-1)	10/55 (18.2%)	1 (1-1)	8/41 (19.5%)	0 (0-0)	0.86 9
Sweating	6/96 (6.3%)	0 (0-0)	3/55 (5.5%)	0 (0-0)	3/41 (7.3%)	0 (0-0)	0.70 9
Facial redness	5/96 (5.2%)	0 (0-0)	2/55 (3.6%)	0 (0-0)	3/41 (7.3%)	0 (0-0)	0.42 2
Neck pain	5/96 (5.2%)	0 (0-0)	2/55 (3.6%)	0 (0-0)	3/41 (7.3%)	0 (0-0)	0.42 2
Blurred vision	5/96 (5.2%)	0 (0-0)	4/55 (7.3%)	0 (0-0)	1/41 (2.4%)	0 (0-0)	0.29 2
Pallor	4/96 (4.2%)	0 (0-0)	4/55 (7.3%)	0 (0-0)	0/41 (0.0%)	0 (0-0)	0.07 8
Ptosis	1/96 (1.0%)	0 (0-0)	1/55 (1.8%)	0 (0-0)	0/41 (0.0%)	0 (0-0)	0.38 5
Myosis	1/96	0 (0-0)	0/55	0 (0-0)	1/41	0 (0-0)	0.24

	(1.0%)		(0.0%)		(2.4%)		4
Mydriasis	0/96<br>(0.0%)	0 (0-0)	0/55<br>(0.0%)	0 (0-0)	0/41<br>(0.0%)	0 (0-0)	1.00<br>0

SUPPLEMENTARY SIII

Supplementary Table S3: Univariate logistic regression of variables associated with the appearance of headache (OR: odds ratio; CI: confidence interval).

**Variable**	**Odds Ratio (OR)**	**95% Confidence Interval**		***P* value**
	**OR**	**Lower end**	**Upper end**	
Sex	1.675	0.684	4.106	0.259
Age	0.968	0.835	1.122	0.668
Headaches usually apart from alcohol-induced headache	2.491	1.079	5.753	**0.033**
Alcohol-induced headache is similar to your usual headaches	0.538	0.212	1.368	0.193
Frequent and severe headaches	3.437	1.347	8.775	**0.010**
Previous hangover (nº indicate the %)	1.622	0.260	10.118	0.604
Usual hours of sleep	1.363	0.902	2.061	0.142
Hours of sleep the night before the episode	1.136	0.831	1.553	0.425
Grams of alcohol during the week	0.957	0.922	0.993	**0.020**
Grams of alcohol on the weekend	0.997	0.992	1.002	0.263
Grams of pure alcohol consumed during the episode	1.001	0.998	1.004	0.541

Consumption of non-alcoholic beverages during the episode	1.176	0.489	2.827	0.718
Quantity in litres of the drink consumed	0.723	0.048	10.988	0.815
Food consumption during the episode	1.464	0.648	3.310	0.360
Types of food consumed	0.984	0.808	1.199	0.875
History of headache in your relatives	0.941	0.413	2.143	0.885
Photophobia	1.673	0.918	3.050	0.093
Phonophobia	1.611	0.674	3.853	0.283
Nausea	1.628	0.555	4.778	0.375
Vomiting	2.156	0.535	8.692	0.280
Cranial-autonomic symptoms (tearing, eye redness, runny nose, nasal fullness)	1.036	0.452	2.377	0.933
Ear fullness	0.741	0.045	12.203	0.834
Asthenia	1.874	0.396	8.874	0.429
Dizziness or vertigo	1.464	0.648	3.310	0.360
Restlessness	1.023	0.411	2.543	0.961
Pallor	******	0.000	*****	0.999
Stiff neck or difficulty moving it	0.478	0.076	3.001	0.431

Blurred vision	3.137	0.337	29.178	0.315
Thirst	0.000	0.000	No sale nada	1.000
Hunger	0.889	0.142	5.579	0.900
Irritability	0.667	0.270	1.649	0.380
Sensitive or any alteration in emotions	0.711	0.300	1.683	0.438
Difficulty thinking or speaking	1.333	0.517	3.441	0.552
Difficulty reading or writing	0.917	0.326	2.574	0.869
Difficulty concentrating	1.775	0.772	4.084	0.177
Preference for lying down	1.134	0.321	4.008	0.845
Sweating	0.731	0.140	3.821	0.710
Flushing or facial redness	0.478	0.076	3.001	0.431
Ptosis	****	0.000	No sale nada	1.000
Miosis	0.000	0.000	No sale nada	1.000
General malaise/abdominal pain/diarrhea	1.775	0.772	4.084	0.177
Duration of phonophobia	1.345	0.844	2.142	0.212
Duration of photophobia	1.656	0.990	2.769	0.055
Duration of osmophobia	1.864	0.864	4.023	0.113
Duration of nausea	1.340	0.705	2.547	0.372
Duration of vomiting	1.797	0.630	5.131	0.273
Duration of cranial-autonomic symptoms (tearing, eye redness,	0.801	0.581	1.104	0.175

runny nose, nasal fullness)				
Duration of ear fullness	0.650	0.232	1.815	0.411
Duration of asthenia	1.245	0.984	1.574	0.068
Duration of dizziness or vertigo	1.379	0.777	2.448	0.272
Duration of restlessness	0.938	0.578	1.521	0.794
Duration of pallor	****	0.0000	No sale nada	0.999
Duration of stiff neck or difficulty moving it	0.478	0.076	3.001	0.431
Duration of blurred vision	0.731	0.304	1.756	0.484
Duration of thirst	1.506	1.086	2.088	**0.014**
Duration of hunger	1.274	0.973	1.669	0.078
Duration of irritability	0.867	0.516	1.457	0.590
Duration of sensitivity or any alteration in emotions	0.803	0.509	1.266	0.345
Duration of the difficulty thinking or speaking	2.566	1.254	5.247	**0.010**
Duration of the difficulty reading or writing	2.830	1.250	6.406	**0.013**
Duration of the difficulty concentrating	1.256	0.824	1.915	0.288
Duration of the preference for lying down	1.191	0.919	1.542	0.187
Duration of sweating	0.534	0.113	2.527	0.429

Duration of flushing or facial redness	0.617	0.156	2.445	0.492
Duration of ptosis	0.370	0.050	2.720	0.328
Duration of miosis	0.000	0.000	No sale nada	0.999
Duration of general malaise/abdominal pain/diarrhea	1.273	0.841	1.926	0.254

Supplementary Figure S1: Amount of pure grams of alcohol within episodes where headache was present versus not present.

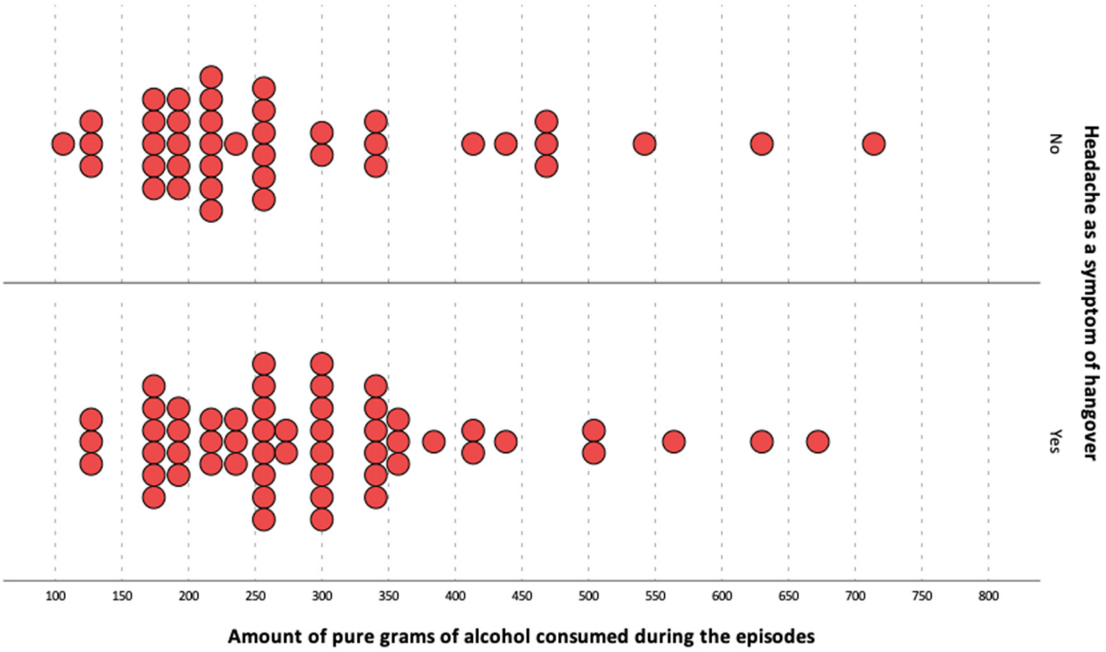

SUPPLEMENTARY SIV

Table S4: Cox regression analysis of the variables associated with a more prolonged duration of the headache.

Variable	Hazard ratio (HR)	95% Confidence Interval		*P* value
	HR	Lower	Upper	
Female sex	0.830	0.439	1.572	0.568
Age	1.022	0.921	1.135	0.676
Occupation	1.560	0.900	2.706	0.113
Usual headache apart from alcohol-induced headache	1.020	0.589	1.765	0.944
Consulted physician for this pain	0.895	0.467	1.717	0.738
Alcohol-induced headache similar to your usual headaches	1.111	0.571	2.162	0.756
Frequent and severe headaches	1.226	0.714	2.103	0.460
Headache duration greater than 4 hours	1.051	0.612	1.807	0.857
Presence of nausea with headache	0.860	0.429	1.722	0.670
Presence of phonophobia or photophobia with headache	0.355	0.107	1.174	0.090
Limitation of physical or intellectual activities	0.700	0.096	5.125	0.726

due to the headache				
Smoker	0.913	0.469	1.775	0.788
Previous hangover (nº indicate the %)	0.735	0.195	2.767	0.649
Usual hours of sleep	1.126	0.847	1.497	0.414
Usual nap	0.793	0.372	1.690	0.548
Hours of sleep the night prior to the hangover	1.057	0.881	1.267	0.553
Grams of alcohol consumed during the week	0.997	0.968	1.027	0.847
Grams of alcohol consumed on the weekend	1.001	0.997	1.005	0.593
Grams of pure alcohol consumed during the episode	1.000	0.998	1.003	0.841
Consumption of non-alcoholic beverages during the episode	1.141	0.643	2.024	0.653
Type of non-alcoholic drink	0.927	0.207	4.146	0.921
Quantity in litres of the drink consumed	0.708	0.133	3.757	0.685
Food intake during the episode	1.366	0.799	2.335	0.255
Types of food consumed	0.973	0.858	1.103	0.665
History of headache in your relatives	0.905	0.522	1.569	0.722
Headache intensity	0.848	0.745	0.964	**0.012**
Headache location	2.820	0.852	9.331	0.090
Time of onset of the headache	0.893	0.497	1.606	0.705
Headache location	0.700	0.096	5.125	0.726
Temporal location	0.989	0.568	1.722	0.969

Parietal location	1.425	0.344	5.901	0.625
Occipital location	4.910	0.634	38.024	0.128
Periocular location	4.910	0.634	38.024	0.128
Pain irradiation	4.910	0.634	38.024	0.128
Quality of the headache	1.164	0.668	2.028	0.592
Stabbing pain	1.141	0.615	2.118	0.675
Throbbing pain	0.457	0.139	1.505	0.199
Pain worsening with head movement	1.025	0.585	1.794	0.932
Pain triggered by standing up	0.663	0.376	1.172	0.157
Pain worsening with standing up	1.354	0.782	2.346	0.280
Pain improvement with supine position	1.636	0.907	2.950	0.102
Worsening of pain	1.354	0.782	2.346	0.280
Pain improvement	1.279	0.953	1.718	0.102
Limitation of physical activities	0.603	0.335	1.087	0.092
Methods used to relieve the pain	0.754	0.559	1.017	0.064
Phonophobia	0.512	0.294	0.892	**0.018**
Photophobia	0.697	0.456	1.065	0.095
Osmophobia	0.752	0.433	1.306	0.312
Nausea	0.765	0.399	1.466	0.420
Vomiting	0.624	0.280	1.391	0.249
Cranial-autonomic symptoms (tearing, eye redness, runny nose, nasal fullness)	1.059	0.607	1.849	0.839

Ear fullness	0.824	0.113	6.006	0.849
Asthenia	0.696	0.215	2.247	0.544
Dizziness or vertigo	0.472	0.264	0.844	**0.011**
Restlessness	1.007	0.552	1.837	0.982
Pallor	0.604	0.217	1.684	0.335
Stiff neck or difficulty moving it	0.873	0.211	3.605	0.851
Blurred vision	0.718	0.258	1.994	0.524
Thirst	0.700	0.096	5.125	0.726
Hunger	0.994	0.308	3.204	0.992
Irritability	0.685	0.359	1.310	0.253
Sensitive or any alteration in emotions	0.811	0.450	1.461	0.486
Difficulty thinking or speaking	0.700	0.384	1.276	0.245
Difficulty reading or writing	0.600	0.299	1.204	0.151
Difficulty concentrating	0.929	0.521	1.655	0.802
Preference for lying down	1.400	0.551	3.558	0.480
Sweating	0.440	0.135	1.442	0.175
Flushing or facial redness	0.873	0.211	3.605	0.851
Ptosis	0.418	0.057	3.073	0.391
General malaise /abdominal pain /diarrhea	0.790	0.447	1.396	0.417
Duration of phonophobia	0.665	0.481	0.921	**0.014**
Duration of photophobia	0.690	0.482	0.990	**0.044**

Duration of osmophobia	0.724	0.476	1.101	0.131
Duration of nausea	0.848	0.592	1.216	0.371
Duration of vomiting	0.771	0.412	1.441	0.415
Duration of cranial-autonomic symptoms (tearing, eye redness, runny nose, nasal congestion)	0.934	0.734	1.189	0.580
Duration of ear fullness	0.647	0.238	1.753	0.391
Duration of dizziness or vertigo	0.669	0.458	0.975	**0.037**
Duration of restlessness	0.944	0.628	1.418	0.780
Duration of pallor	0.604	0.217	1.684	0.335
Duration of stiff neck or difficulty moving it	0.873	0.211	3.605	0.851
Duration of blurred vision	0.718	0.258	1.994	0.524
Duration of thirst	0.905	0.737	1.112	0.343
Duration of hunger	0.953	0.810	1.121	0.564
Duration of irritability	0.952	0.593	1.527	0.837
Duration of sensitivity or any alteration in emotions	0.853	0.564	1.290	0.451
Duration of difficulty thinking or speaking	0.705	0.471	1.056	0.090
Duration of difficulty reading or writing	0.634	0.404	0.997	**0.048**
Duration of difficulty concentrating	0.865	0.643	1.164	0.338
Duration of preference for lying down	0.880	0.737	1.050	0.155
Duration of sweating	0.440	0.135	1.442	0.175

Duration of redness	1.004	0.387	2.604	0.993
Duration of ptosis	0.418	0.057	3.073	0.391
Duration of general malaise / abdominal pain / diarrhea	0.889	0.668	1.184	0.422

SUPPLEMENTARY SV:

Supplementary Table S5: Multivariate Cox regression analysis: variables associated with headache duration (HR: Hazard Ratio; CI: Confidence Interval).

**Variable**	**Hazard ratio (HR)**	**95% Confidence Interval**	***P* value**
Occupation	1.527	0.515-4.534	0.445
Presence of photophobia or phonophobia with the headache	0.492	0.089-2.723	0.417
Headache intensity	0.833	0.662-1.048	0.119
Pain location	1.328	0.142-12.381	0.803
